# Aphasic Status Epilepticus Treated in the Emergency Room: A Report of Two Cases

**DOI:** 10.7759/cureus.98642

**Published:** 2025-12-07

**Authors:** Yuichiro Inatomi, Makoto Nakajima, Toshiro Yonehara

**Affiliations:** 1 Neruology, Saiseikai Kumamoto Hosiptal, Kumamoto, JPN; 2 Neurology, Graduate School of Medical Sciences, Kumamoto University, Kumamoto, JPN; 3 Neurology, Saiseikai Kumamoto Hospital, Kumamoto, JPN

**Keywords:** aphasia, aphasic status epilepticus, epilepsy, non-convulsive status epilepticus, stroke mimics

## Abstract

The clinical characteristics of patients with an aphasic status epilepticus (ASE) remain unclear. Here, we present two cases with ASE. Case 1 was a 61-year-old man who was admitted to our hospital with cortical deafness and severe aphasia. He was treated in our emergency department for status epilepticus, and his symptoms resolved. Electroencephalography (EEG) showed lateralized periodic discharges in the left temporo-parietal lobes. Single-photon emission computed tomography (SPECT) showed a high-uptake lesion in the left temporo-parietal lobes. Case 2 was an 80-year-old woman who was admitted to our hospital with tonic-clonic seizures. Even after she became alert, she continued to have severe aphasia. EEG showed generalized spike-and-slow wave complexes. She was treated for status epilepticus, and her symptoms resolved. SPECT after her aphasia resolved showed high-uptake lesions in both parietal lobes. At the time of her recurrence 2.5 years later, she was treated in our emergency department for status epilepticus, and her symptoms immediately resolved. Both cases were diagnosed with aphasic status epilepticus. ASE should be considered as a stroke mimic and may cause sequelae due to delayed treatment. We recommend that patients suspected of ASE should be treated immediately with benzodiazepine without waiting for electroencephalography or perfusion imaging when ischemic stroke is ruled out by MRI, including diffusion-weighted imaging (DWI).

## Introduction

Non-convulsive status epilepticus (NCSE) represents a prolonged status of seizure (status epilepticus (SE)) without marked motor manifestations [1]. NCSE with aphasia is called aphasic status epilepticus (ASE) and has been referred to separately since its first description by de Pasquet et al. (1976) [2]. Rosenbaum et al. (1986) and Grimes et al. (1997) defined ASE as follows: 1) the patient must have language production (“speaking” in Rosenbaum’s article) during seizures; 2) language production must show aphasic features (Rosenbaum suggested dysfluency, dysnomia, or paraphasia as examples of aphasia); 3) consciousness must be preserved; 4) the seizures must be correlated with the aphasia, as documented by electroencephalography (EEG) and behavioral testing; and 5) (added by Grimes) the aphasia should resolve, or nearly so, concurrent with the successful treatment of the seizures [3, 4]. Since the publication of these reports, ASE has been diagnosed based on this definition and has been described in many case reports and case series reports [5-8].

As stated in the definition above, an EEG is required to diagnose ASE, although it is believed that many medical facilities are unable to perform an EEG on a full-time basis. Thus, there is a concern about delays in diagnosis and treatment for patients with ASE. However, the clinical characteristics and prognosis of patients with ASE are still unclear.

In this article, we present two cases with ASE. Both patients were diagnosed immediately with ASE and treated in the emergency room, resulting in complete remission within their acute phase.

## Case presentation

Case 1

This patient is the same as one of the patients in our previous case report [9]. In republishing this case report, we have added detailed descriptions of the patient's clinical history and findings, including the disturbance of consciousness, language symptoms, and recall of episodic memories during the ASE.

A 61-year-old, right-handed man entered a convenience store at midnight and stated repeatedly “*mimi-gakikoenai*” (*I cannot hear*) and “*zatsuon-ga suru*” (*I am hearing noise*). He was transported to the emergency room of our hospital. He had a history of brain infarcts in the right lateral temporal lobe at 57 years of age and the left medial temporo-occipital lobes at 59 years of age; thereafter, he had right upper homonymous quadrantanopia and mild amnesia. Two months after the onset of the second brain infarct, the patient experienced a simple visual hallucination of a sphere or spark. He was diagnosed with epilepsy, for which he had started taking 500 mg of levetiracetam tablets. He did not experience difficulties in conversation or daily life.

Upon examination at 50 minutes after onset, the patient could open his eyes spontaneously and when other people touched him. However, he did not open his eyes when the examiner made a loud noise. He had normal muscle power and a sense of pain. When the patient’s eyes were open, he demonstrated bilateral visual-spatial attention and was able to imitate several of the examiner’s poses. He spoke only the two sentences mentioned above. He had difficulties in repetition, naming, responding to questions, and obeying verbal and written commands. The patient was diagnosed with global aphasia with recurrent utterances (i.e., the two sentences he could say) and cortical deafness.

**Figure 1:**
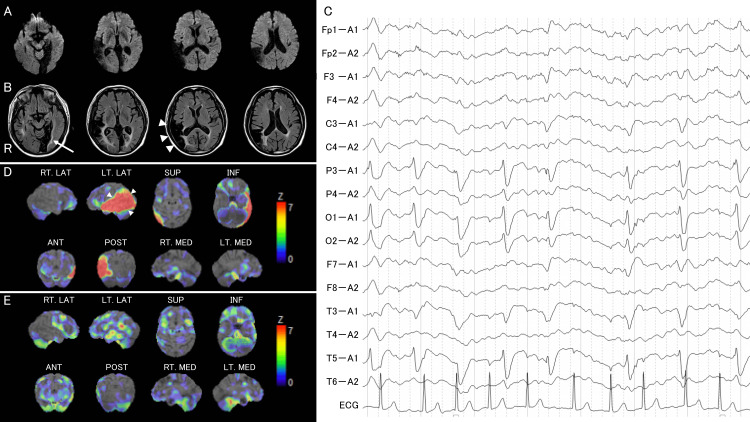
Neuroimaging of Case 1 A: Diffusion-weighted imaging two hours after onset revealing no new lesions. B: Fluid-attenuated inversion recovery imaging showing old infarcts in the right lateral temporo-parietal lobes that had spread to the transverse temporal gyrus (arrowheads) and subcortical white matter, including the acoustic radiation, and left medial temporo-occipital lobes (arrow). C: Electroencephalography at 10 hours after onset, revealing 1 Hz lateralized periodic discharges in the left parieto-temporal region. D: Single-photon emission computed tomography on Day 1, revealing a high-uptake lesion in the left superior, middle, and inferior temporal gyri and inferior parietal lobule (arrowheads). E: Follow-up single-photon emission computed tomography on Day 7 showing the disappearance of the high-uptake lesion that was observed on admission.

Magnetic resonance imaging (MRI), including diffusion-weighted imaging (DWI), at two hours after onset (Figure 1A) revealed no new lesions, including brain infarcts. Fluid-attenuated inversion recovery (FLAIR) imaging showed the old infarcts in the right lateral temporal lobe that had spread to the transverse temporal gyrus and subcortical white matter, including the acoustic radiation, and the left medial temporo-occipital lobes (Figure 1B). There were no signs of cerebral arterial occlusion or hyperintense vessel signs on the FLAIR imaging, nor gradient-echo susceptibility vessel signs on the T2-weighted imaging.

Since it was determined that these neurological deficits were caused by NCSE, we administered 10 mg intravenous diazepam and a drip infusion of 250 mg phenytoin sodium immediately after MRI. However, his aphasia and deafness persisted. Therefore, additional phenytoin was administered six hours after onset, after which the patient slept immediately. When he awoke at 10 hours after onset, he could answer his name correctly and obey simple commands, although he was unable to name objects and repeat their names. He was treated with 1,000 mg of levetiracetam. EEG obtained 10 hours after onset (Figure 1C) showed 1-Hz lateralized periodic discharges in the left parieto-temporal region. On Day 1, he was able to engage in an easy conversation, although phonemic paraphasia and difficulty in word-finding and repetition of sentences were still observed in his speech. Single-photon emission computed tomography (SPECT) on Day 1 (Figure 1D) revealed a high-uptake lesion in the left superior, middle, and inferior temporal gyri and inferior parietal lobule.

On Day 5, when his speech ability seemed to have recovered to its pre-onset state, he described his experience at onset as, “When I was watching the television at home, I realized that I could not understand any of the words spoken on it. I could also hear a noise like a radio. I understood that I had been taken to a hospital, but I could not speak or listen.” On Day 5, no epileptic discharges were observed on the EEG. The high-uptake lesion was no longer present on SPECT on Day 6 (Figure 1E). The patient was discharged from the hospital on Day 7.

Two years later, the patient experienced an attack similar to the original episode. He was admitted to the emergency room of our hospital again. Neurological examination on admission revealed global aphasia and cortical deafness, and DWI revealed no new lesions. Therefore, he was treated with 20 mg intravenous diazepam one hour after onset. When he awoke four hours after onset, he could engage in an easy conversation, although phonemic paraphasia and difficulty in word-finding and repetition were observed. On Day 2, his aphasia and deafness were resolved completely, and an EEG revealed lateralized periodic discharges in the left parieto-temporal region. He was treated with 1,200 mg of gabapentin in addition to levetiracetam. The patient was discharged from our hospital on Day 14. At seven years since the last attack, he has been free from brain infarcts and epileptic episodes and is independent in his daily life.

Case 2

**Figure 2:**
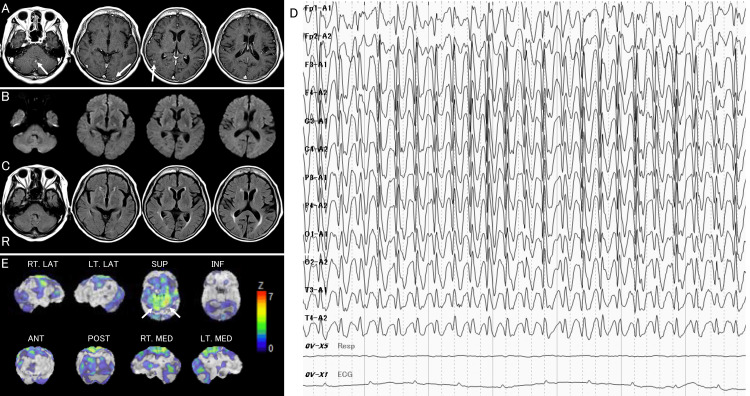
Neuroimaging of Case 2 at the first attack A: T1-weighted imaging four months before the first attack revealing well-enhanced lesions in the cerebellar vermis and bilateral occipital lobes, indicating metastatic tumors (arrows). B: Diffusion-weighted imaging 22 hours after onset, revealing no new lesions. C: Fluid-attenuated inversion recovery imaging at 22 hours after onset showing no lesions, including metastatic lesions, which were observed four months ago. D: Electroencephalography at 22 hours after onset, revealing spike and wave complex in the bilateral whole brain. E: Single-photon emission computed tomography on Day 5, revealing a high-uptake lesion in the bilateral superior and inferior parietal lobules (arrows).

An 80-year-old, right-handed woman was transported to the emergency room of our hospital due to a tonic-clonic seizure lasting a few seconds. She had experienced a loss of consciousness several times and was diagnosed with epilepsy, for which she was prescribed 1,000 mg levetiracetam. She was also diagnosed with lung adenocarcinoma four months earlier and was treated with osimertinib. At the same time, she was also diagnosed with metastatic brain tumors in the right cerebellar hemisphere and left occipital lobe on MRI (Figure 2A). She did not experience difficulties in conversation or daily life.

On admission at 80 minutes after onset, she could open her eyes spontaneously and had normal muscle power and sense of pain. She spoke several words only, including “*hai*” (*yes*), “*konnichiwa*” (*hello*), “*yokadesu*” (*I feel good*), and ”*itai*” (*ouch*). She had difficulties in repetition, naming, responding to questions, and obeying verbal or visual commands, visual gestures, and pantomime commands. She was also able to imitate several of the examiner’s poses.

Brain computed tomography (CT) revealed no new lesions. She was treated with 500 mg intravenous levetiracetam at our emergency room; thereafter, she did experience a “convulsive” seizure. On the morning of Day 1, she could still utter the same words as on admission, although she still had difficulty with repetition, verbal comprehension, and naming. However, she had a good understanding of the situation and was able to eat with chopsticks, get dressed, walk to the toilet, and perform excretory activities. DWI at 22 h after onset (Figure 2B) revealed no new lesions, including brain infarcts. FLAIR imaging showed no lesions, including metastatic lesions, which were observed 4 months earlier. (Figure 2C). There were no signs of cerebral arterial occlusion or hyperintense vessel signs or susceptibility vessel signs. EEG at 22 hours after onset (Figure 2D) revealed spike and wave complexes in the bilateral whole brain. It was determined that her aphasia was caused by NCSE. Therefore, we administered 5 mg intravenous diazepam; thereafter, immediately and without falling asleep, she was able to state her name and the name of our hospital and obey some simple commands correctly. She was treated with 1,500 mg of levetiracetam.

On Day 2, she did not have any difficulty in simple daily conversation; however, she had difficulty in mild verbal comprehension, naming, and word-finding, and phonemic and verbal paraphasia were observed in her speech. At that time, she could describe her experience of the events that occurred on Day 1, including her difficulty in speech, clinical consultation, rehabilitation, and undergoing an EEG and MRI. However, she did not remember any events on Day 0. On Day 4, no epileptic discharges were observed on the EEG. By Day 4, her aphasia was completely resolved. SPECT on Day 5 revealed a high-uptake lesion in the bilateral superior and inferior parietal lobules (Figure 2E). She was transferred to a rehabilitation hospital on Day 6. She was discharged from that hospital one month later and has since resumed living alone.

**Figure 3:**
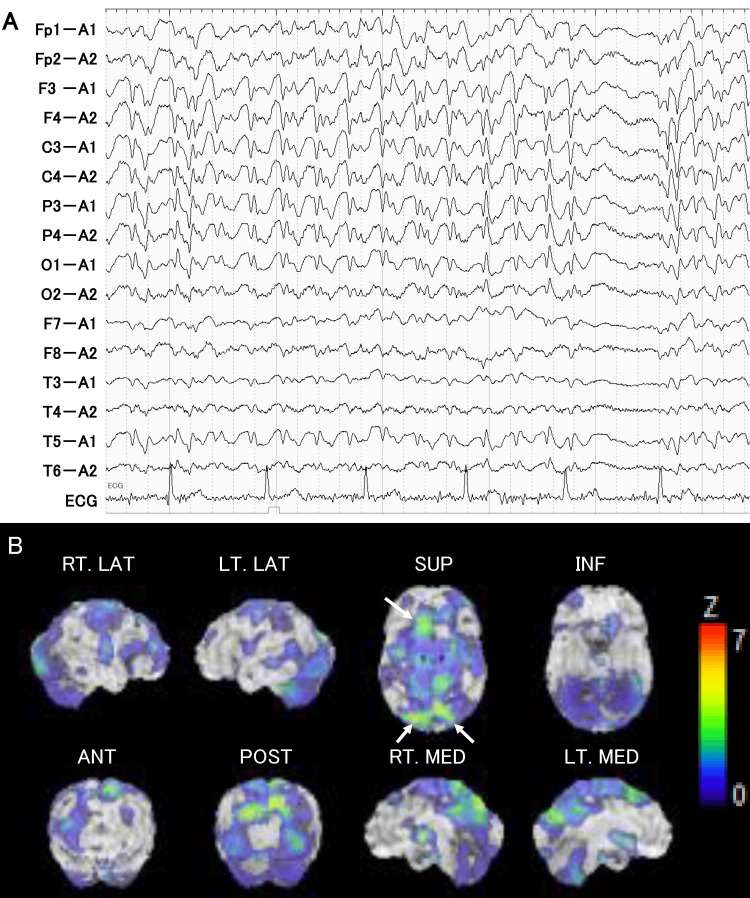
Neuroimaging of Case 2 at the second attack A: Electroencephalography six hours after onset, revealing spike and wave complex in the bilateral fronto-parieto-occipital lobes. B: Single-photon emission computed tomography on Day 1 revealing a high-uptake lesion in the bilateral superior parietal lobules (arrows) and left superior and middle frontal gyri (arrow).

At 2.5 years post onset, she was readmitted to the emergency room of our hospital due to difficulty in speech without convulsive seizures. Neurological examination on admission at 90 minutes after onset revealed global aphasia with only a few words. On the basis of a diagnosis of NCSE, she was treated quickly with 5 mg intravenous diazepam without waiting for neuroradiological imaging, and she was able to speak immediately. DWI revealed no new lesions, including brain infarcts. EEG obtained six hours after onset showed spike and wave complexes in the bilateral fronto-parieto-occipital lobes (Figure 3A). SPECT on Day 1 revealed a high-uptake lesion in the bilateral superior parietal lobules and left superior and middle frontal gyri (Figure 3B). She was treated with 100 mg of lacosamide in addition to levetiracetam. On Day 2, her conversation improved, with verbal paraphasia and difficulty in comprehending long sentences. EEG on Day 3 showed no epileptic discharges. At that time, she was able to describe her experience at onset, including a sudden difficulty in speech and transport by ambulance, although she did not remember any events in the emergency room on Day 0. By Day 4, her aphasia was completely resolved, and she was discharged from our hospital on Day 8. Three years after the last attack, she has been free from brain infarcts and epileptic episodes, as well as the recurrence of lung cancer and brain metastasis, and is independent in her daily life.

## Discussion

In both present cases, even immediately after their attacks, when their verbal impairments were severe, the patients had good activity and attention to their surroundings and were able to walk and eat. Moreover, after language recovery, they could recall the events during their verbal impairments. Therefore, it was considered that their consciousness was clear during this period. In addition, their verbal impairments, including difficulties in hearing comprehension and word-finding, and phonemic or verbal paraphasia during the recovery process, indicated the characteristics of aphasia. Epileptic discharges were observed on EEG during the presence of aphasia, and their aphasia improved immediately with the administration of antiepileptic agents. On the basis of their clinical courses, both cases were considered to meet the definition of ASE proposed by Rosenbaum et al. (1986) and Grimes et al. (1997) [3, 4].

We expected that the epileptic focus would be limited to the language area. However, in Case 2, epileptic findings were observed in the bilateral cerebral hemispheres. In two reported cases [10, 11], epileptic findings were observed in bilateral cerebral hemispheres, as in our Case 2. In studies using subdural electrode electrical stimulation, aphasia could be induced even with the stimulation of a limited area [12, 13]. Therefore, it is suggested that cortical epileptic activity limited to the language area alone is sufficient to cause ASE; however, this limited intracranial epileptic activity may be amplified, and epileptic discharges may be detected over a wider area in EEG using extracranial electrodes.

Conversely, epileptic findings on EEG may be underestimated in patients with ASE. Ericson et al. (2011) reported that in nine patients with ASE, epileptic discharges were observed in only five during a 30-minute EEG, even during the aphasic state; however, they were detected in all of the patients using continuous video EEG [14]. Moreover, there was a case in which NCSE was diagnosed with DWI and arterial spin labeling perfusion MRI without the detection of epileptic discharges on EEG [15]. Therefore, we believe that there are patients with ASE in whom the responsible lesions estimated from their aphasia are not compatible with the epileptic focus identified from the EEG.

In a reported case series study of ASE, hyperperfusion in the region associated with epilepsy on perfusion imaging was observed in five of eight patients [5]. Conversely, perfusion imaging was performed over a period of more than two weeks after the onset of ASE, in which hypoperfusion was observed in the region associated with epilepsy [16, 17]. Furthermore, in cases that underwent serial perfusion imaging, there was a decrease in blood flow [16, 18], and similar changes were observed in our Case 1. Therefore, in the reported cases of ASE with hypoperfusion in the epileptic lesions, it is suggested that perfusion imaging was performed long after the onset of ASE.

Both our patients were diagnosed immediately with ASE and were treated in the emergency room, resulting in a complete remission in the acute phase. However, as discussed earlier, it is expected to be difficult to make a rapid diagnosis in patients with ASE, because clinical information supporting SE and conclusive neuroradiological imaging findings are often lacking. Indeed, there are cases of ASE that took four months [19] and two months [20] from onset to treatment. Jaraba Armas et al. (2022) also stated that there were 28% of cases in which ASE improved within one day, and also reported the following outcomes for patients with ASE: return to baseline in 48%, no return to baseline in 36%, and death in 16% [8].

Conversely, it is problematic to determine whether a patient is alert, as required by the definition, when questioning is difficult due to the presence of aphasia. In previous reports of ASE, there has been little discussion about whether a patient was alert or not. Moreover, in our review of ASE cases, convulsive seizures were present, or there was a history of epilepsy. In such cases, there is a risk of the misdiagnosis of a prolonged communication disorder simply due to the presence of post-epileptic drowsiness.

As in both of our patients, it is suggested that strong evidence for determining whether a patient is alert is provided by the fact that “even during a communication disorder, the patient is presumed to have maintained spontaneity and attention to the outside world through eating, walking, and nonverbal communication” and “if verbal communication is possible after recovery, the patient is able to recall events that occurred during their communication disorder.”

As mentioned above, ASE should be considered as a stroke mimic, and delayed treatment may cause sequelae. Therefore, patients with ASE should be treated immediately with antiseizure agents, including intravenous benzodiazepine. It is expected that the accuracy of ASE diagnosis will be improved by using multiple neurofunctional imaging approaches in addition to clinical consultations. However, there are a certain number of cases in which a definitive diagnosis is difficult, even with the use of this neurofunctional imaging. We recommend that patients suspected of having ASE should be treated quickly with benzodiazepine without waiting for EEG and perfusion imaging.

## Conclusions

We presented two cases with ASE. Their consciousness was clear during the aphasic attack. Epileptic discharges were observed on EEG during the presence of aphasia, and their aphasia improved immediately with the administration of antiepileptic agents. Moreover, at the time of their recurrence, both patients were diagnosed immediately with ASE and were treated in the emergency room, resulting in a complete remission in the acute phase. ASE should be clinically considered as a stroke mimic, and delayed treatment may cause sequelae. We recommend that patients suspected of having ASE should be treated quickly with benzodiazepine without waiting for EEG and perfusion imaging when ischemic stroke is ruled out by MRI, including DWI.
